# Toothache—Systemic Treatment

**Published:** 1873-04

**Authors:** 


### Toothache—Systemic Treatment.

Dr. A. L. Cory, (*Chicago Med. Times,*) "reports a new remedy for toothache. Being called upon immediately after the fire, to extract some aching teeth, and not having the necessary implements, and most of the dentists having lost their instruments, he prescribed 50 grains of hydrate chloral, to lessen the pain and produce rest until he could obtain the necessary forceps. To his astonishment, in every case, the ache not only promptly vanished, but failed to return. The Doctor says he has now tried this plan in about twenty cases with quite uniform success."

Various anodynes will answer the same purpose. Among others, ionoform in one grain doses is a very efficient remedy for dental and facial neuralgia.—*Medical Cosmos.*

